# Clinical Profiles and Reasons for Emergency Department Presentation Among Oncology Patients—A Retrospective Two-Center Study in Poland

**DOI:** 10.3390/jcm15083090

**Published:** 2026-04-17

**Authors:** Anna Ingielewicz, Zuzanna Brunka, Mariusz Grażewicz, Mateusz Szczupak, Marzena Szarafińska, Robert K. Szymczak

**Affiliations:** 1 Department of Emergency Medicine, Medical University of Gdańsk, Ul. M. Sklodowskiej-Curie 3a, 80-210 Gdansk, Polandrobert.szymczak@gumed.edu.pl (R.K.S.); 2 Department of Emergency Medicine, University of Warmia and Mazury, ul. M. Oczapowskiego 2, 10-719 Olsztyn, Poland; 3 Szpital im. Mikołaja Kopernika, ul. Nowe Ogrody 1-6, 80-803 Gdańsk, Poland

**Keywords:** palliative medicine, emergency medicine, oncology patients, cancer-related emergencies, end-of-life care, healthcare utilization

## Abstract

**Background/Objectives**: Cancer patients increasingly present to emergency departments, posing unique clinical and organizational challenges. Data on this population in Poland remain limited. **Methods**: A retrospective study was conducted in two hospitals in northern Poland (January–March 2023). All adult patients with active cancer presenting to the ED were included (n = 552, 3.1% of visits). Data included demographics, cancer type, presenting complaints, Emergency Severity Index (ESI), disposition, and in-hospital mortality. Multivariable logistic regression models were used to assess predictors of hospitalization, hospice referral, and mortality, reported as odds ratios (ORs) with 95% confidence intervals (CIs). **Results**: Mean age was 68 years; 51% were female. The most common cancers were lung, breast, colorectal, and prostate. Leading complaints included abdominal pain (15%), trauma (7.5%), and dyspnea (7%). Most patients were triaged as ESI 3–4 (87%). Hospitalization rate was 58%, hospice referral 6%, and in-hospital mortality 7.1%. Lower ESI levels were significantly associated with hospitalization (OR 0.57; 95% CI 0.44–0.73), hospice referral (OR 0.40; 95% CI 0.25–0.63), and in-hospital mortality (OR 0.29; 95% CI 0.18–0.47). **Conclusions**: Oncology patients represent a small but high-risk ED population. While ESI reflects acute severity, it may not adequately capture palliative care needs. These findings suggest opportunities to improve integration of palliative care in ED settings.

## 1. Introduction

Over recent decades, emergency departments (EDs) worldwide have experienced a sustained increase in patient volume, including a growing proportion of individuals living with active cancer. Advances in oncological therapies have improved survival and, in many cases, transformed cancer into a chronic condition. Consequently, a larger population of patients with complex comorbidities, treatment-related toxicities, and progressive disease trajectories increasingly relies on acute care services [1,2,3].

Patients with cancer account for approximately 3–6% of all ED visits but exhibit disproportionately high rates of hospitalization, intensive care utilization, and short-term mortality [1,4]. Common reasons for ED presentation include pain, dyspnea, infection, thromboembolic events, metabolic disturbances, treatment-related complications, and oncologic emergencies such as spinal cord compression or superior vena cava syndrome [2,5]. In addition, trauma and exacerbations of non-oncologic chronic diseases further contribute to ED utilization in this population [6].

For patients with advanced malignancies, the ED often functions as a critical safety net and, in many cases, one of the final points of contact with the healthcare system [7,8]. In this time-sensitive and high-pressure environment, clinicians must make rapid decisions that can significantly influence the trajectory of care. However, emergency medicine is traditionally oriented toward acute stabilization and life-prolonging interventions, which may not always align with patient preferences, goals of care, or prognostic realities at the end of life [9,10].

Despite the growing integration of palliative care into healthcare systems, substantial barriers to its early implementation in emergency settings persist. Emergency clinicians frequently report limited confidence in recognizing terminal disease trajectories, initiating goals-of-care discussions, or facilitating hospice referrals [9]. Structural constraints—such as time pressure, limited access to longitudinal medical data, and lack of standardized screening tools—further complicate the identification of patients with unmet palliative needs [11,12].

Triage systems play a central role in emergency care by prioritizing patients based on urgency and anticipated resource utilization. The Emergency Severity Index (ESI), widely used internationally, has demonstrated validity in predicting resource use, hospitalization, and mortality, including among oncology patients [13,14,15,16]. However, acuity-based triage tools are not designed to capture key dimensions of palliative care, such as functional decline, symptom burden, psychosocial distress, or discordance between treatment intensity and patient goals [10,17].

As a result, patients with advanced cancer and significant palliative needs may remain under-recognized within existing emergency care frameworks, despite substantial symptom burden and limited life expectancy [7,10,12]. Understanding patterns of ED presentation, triage classification, and clinical outcomes in this population is therefore essential for identifying opportunities to better integrate palliative care into acute care pathways.

Data on emergency department utilization among oncology patients in Central and Eastern Europe remain limited, particularly in healthcare systems where palliative care integration is still evolving [3,4]. This gap restricts the ability to compare international patterns and to identify system-level improvements tailored to regional contexts.

This study aimed to characterize emergency department presentations of adult oncology patients in two Polish hospitals, with a focus on clinical profiles, triage categorization, hospitalization, hospice referral, and in-hospital mortality, in order to identify potential gaps in end-of-life care delivery.

## 2. Methods

### 2.1. Study Design and Setting

This retrospective observational study was conducted in two tertiary hospitals in northern Poland between 1 January and 31 March 2023. This period was selected to reflect peak ED utilization; however, seasonal effects may limit generalizability.

Emergency Department 1 (ED1) was located at Nicolaus Copernicus Hospital in Gdańsk, Poland, a full-profile academic hospital providing outpatient oncology services and day chemotherapy units but without an in-house palliative care consultation team within the ED.

Emergency Department 2 (ED2) was located at the Ministry of Interior and Administration (MSWiA) Hospital in Olsztyn, Poland, a regional oncology center providing chemotherapy, radiotherapy, and hematology services. Similar to ED1, no structured or routine palliative care consultations were available directly within the emergency department during the study period.

Both EDs operate within the Polish public healthcare system and use the Emergency Severity Index (ESI) as the standardized triage tool.

### 2.2. Study Population

All adult patients (≥18 years) with active cancer were included. Repeated visits by the same patient were allowed and treated as independent encounters.

Active cancer was defined as:

- Histologically or clinically confirmed malignancy,
- Ongoing oncologic treatment (chemotherapy, radiotherapy, immunotherapy, hormonal therapy),
- Documented advanced or metastatic disease under active medical follow-up.

All adult patients (≥18 years) with active cancer presenting to either ED during the study period were screened for inclusion.

### 2.3. Data Collection

Presenting complaints were derived from ED documentation and grouped into clinically meaningful categories by three independent investigators trained in standardized data abstraction procedures. A predefined data collection template was used to ensure consistency. In cases of uncertainty or incomplete entries, records were reviewed jointly to reach consensus.

Cancer types and symptoms were categorized using predefined groupings.

Missing data were handled using complete-case analysis; missingness was low (<5% for all variables).

All data were anonymized prior to statistical analysis in accordance with data protection regulations.

The following variables were collected:

- Demographics: age, sex.
- Oncological diagnosis: ICD-10 codes and categorized cancer type.
- Presenting complaint (based on ICD-10 classification).
- Emergency Severity Index (ESI) triage level (from 1 to 5).
- Disposition decision (discharge, hospital admission, hospice referral).
- Hospital ward of admission.
- In-hospital mortality (death in ED or during subsequent hospitalization).

Hospice referral was defined as documented transfer or discharge planning directly involving hospice services.

### 2.4. Outcomes

Primary outcomes included hospital admission, hospice referral, in-hospital mortality.

Secondary outcomes included distribution of ESI triage levels, association between triage level and clinical outcomes.

### 2.5. Statistical Analysis

Statistical analyses were performed using Python (version 3.10.12) with the following libraries: pandas (version 2.2.0), scipy (version 1.11.4), statsmodels (version 0.14.4), and matplotlib (version 3.8.2). Descriptive statistics were used to summarize baseline characteristics. Categorical variables were expressed as counts and percentages. Continuous variables were presented as mean ± standard deviation (SD). Results of regression analyses were reported as odds ratios (ORs) with 95% confidence intervals (CIs).

Comparisons between categorical variables were conducted using Chi-square (χ^2^) tests. Associations between ESI triage levels and outcomes were assessed using both bivariate analysis and multivariable logistic regression models.

Three separate logistic regression models were constructed to evaluate predictors of hospital admission, hospice referral, and in-hospital mortality.

Independent variables included age (continuous), sex (binary), cancer type (categorical), presenting symptom category, and ESI triage level.

Odds ratios (ORs) with 95% confidence intervals (CIs) were calculated.

A two-sided *p*-value < 0.05 was considered statistically significant.

### 2.6. Ethical Considerations

The study protocol was approved by the Independent Bioethics Committee of the University of Warmia and Mazury (approval number NKBUWM 2/2025 issued on 20 February 2025). Due to the retrospective and anonymized nature of the study, the requirement for informed consent was waived in accordance with national regulations.

## 3. Results

During the study period, 567 ED visits involving patients with a documented oncological diagnosis were identified. After exclusion of 15 cases due to incomplete documentation or lack of active disease, 552 patients were included in the final analysis.

Of all patients admitted to the emergency department during the study period (n = 18,017), 552 people with cancer were included in the analysis, accounting for 3.1% of all admissions. In the study group, women accounted for 51% (n = 280) and men for 49% (n = 272); the average age was 68.4 years (SD 13.0). Table 1 presents a comparison of the two emergency departments in terms of the main clinical outcomes.

The most common oncological diagnoses included lung, breast, colorectal, and prostate cancer (C34, C50, C18, C61). The distribution of oncological diagnoses is presented in Table 2.

**Figure 1:**
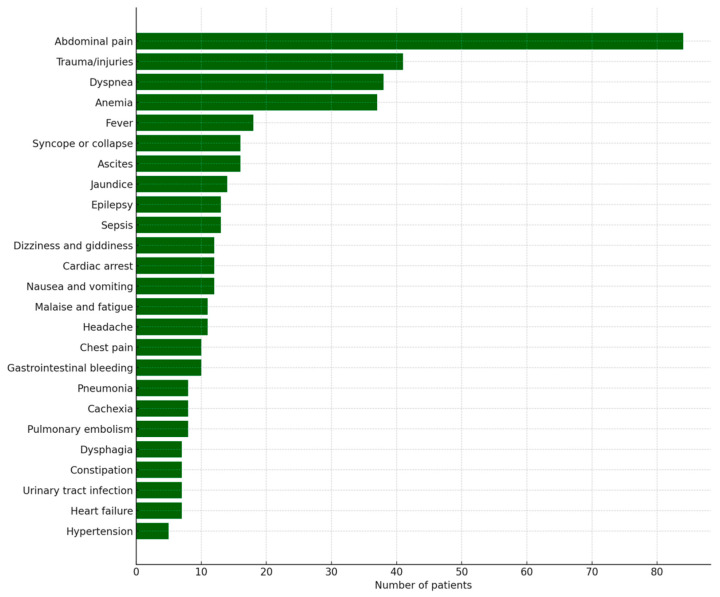
Most frequent presenting symptoms among oncology patients admitted to the Emergency Department. Symptoms were grouped by ICD-10 codes and include trauma-related conditions.

The main complaints leading to visits to the emergency department were abdominal pain (15%), injuries (7.5%), shortness of breath (7%), and anemia (6.9%). The most frequently reported complaints are presented in Figure 1.

**Figure 2:**
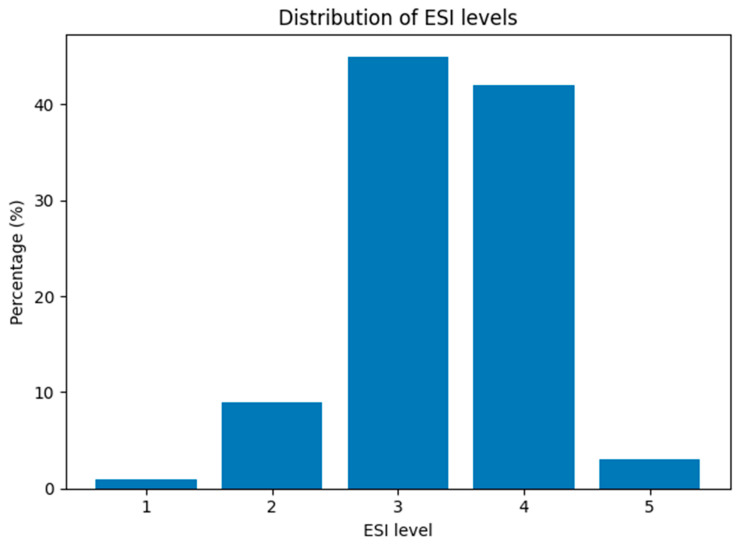
Distribution of ESI triage levels among oncology patients presenting to the ED.

An ESI triage level of 3 or 4 was recorded in over 87% of patients; the detailed percentage distribution is shown in Figure 2.

Only 6% of patients (n = 34) received a referral to palliative care (home or inpatient hospice) in the emergency department or hospital, most of whom were hospitalized patients.

**Figure 3:**
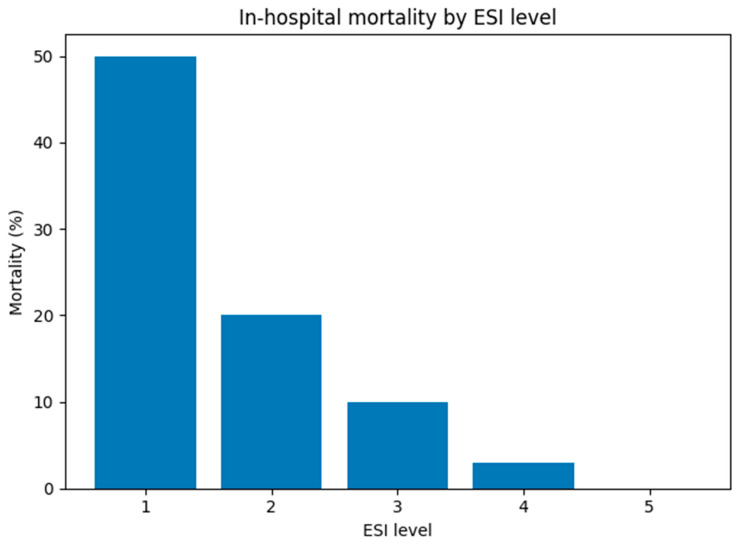
Association between ESI triage levels and in-hospital mortality among oncology patients presenting to the ED. Data are shown as percentage of deaths within each triage level. A statistically significant association was found (*p* < 0.0001).

During hospitalization, 39 patients died, accounting for 7.1% of the study group. The association between in-hospital mortality and the ESI triage level was statistically significant. Results of multivariable logistic regression analyses are presented below (Figure 3).

Multivariable logistic regression analyses confirmed that lower ESI triage level was independently associated with all major outcomes. Lower ESI level was significantly associated with increased likelihood of hospital admission (OR 0.57; 95% CI 0.44–0.73; *p* < 0.001). Older age was also a significant predictor of hospitalization (OR 1.02 per year; 95% CI 1.00–1.03; *p* = 0.023), while sex was not significantly associated. For hospice referral, lower ESI level remained the strongest predictor (OR 0.40; 95% CI 0.25–0.63; *p* < 0.001), with no significant associations observed for age or sex. In-hospital mortality was strongly associated with lower ESI levels (OR 0.29; 95% CI 0.18–0.47; *p* < 0.001), while age and sex were not statistically significant predictors.

The magnitude and consistency of the ESI effect across all models indicate a strong and clinically meaningful association with patient outcomes.

## 4. Discussion

This study demonstrates that although oncology patients constitute a relatively small proportion of emergency department (ED) visits, they represent a clinically high-risk population with substantial healthcare needs. High rates of hospitalization and in-hospital mortality, combined with low rates of hospice referral, suggest that the ED frequently serves as a site of care for patients with advanced or end-of-life disease without adequate integration of palliative principles [1,2,4].

A key finding of this analysis is the limited recognition of end-of-life trajectories in the emergency setting. While the Emergency Severity Index (ESI) proved to be a strong predictor of hospitalization and mortality, it primarily reflects acute physiological instability and anticipated resource utilization rather than broader dimensions of palliative need. Consequently, patients with progressive functional decline, refractory symptom burden, advanced cancer-related complications, or misalignment between treatment intensity and goals of care may remain under-recognized from a palliative perspective, despite significant clinical vulnerability [15,16].

The low rate of hospice referral observed in this cohort is consistent with prior studies describing missed opportunities for timely palliative care integration in emergency departments [7,8,12]. Emergency clinicians often operate under time pressure, with limited access to longitudinal patient data and uncertainty regarding prognosis [9]. In such contexts, clinical decision-making may favor hospital admission and diagnostic escalation, even when these interventions provide limited benefit at the end of life [10,11]. Without structured palliative support, ED care may inadvertently contribute to burdensome or non-beneficial interventions during the terminal phase of illness [8].

Importantly, the patterns observed in this study are not unique to the Polish healthcare system. Similar findings—including high ED utilization, frequent hospital admissions, and limited palliative care involvement—have been reported across diverse healthcare settings [7,10,12]. These results therefore reflect a broader, internationally relevant gap at the interface between emergency medicine and palliative care.

Several potential strategies may help address these challenges. First, the implementation of ED-based palliative screening tools may facilitate earlier identification of patients with advanced disease, high symptom burden, or unmet supportive care needs. Second, improved access to palliative care consultation—either on-demand or via predefined clinical triggers—may support timely goals-of-care discussions and more appropriate disposition decisions. Third, the development of structured referral pathways from the ED to hospice or community-based palliative services may reduce barriers to end-of-life care. Finally, enhanced education and training in palliative care principles for ED clinicians may improve confidence in prognostication, communication, and decision-making in complex end-of-life scenarios.

The findings of this study should be interpreted in the context of several limitations. First, the three-month study period may overrepresent winter-related presentations, particularly those associated with infectious complications, and may therefore limit generalizability to other seasons. Second, the retrospective design limited the availability of potentially important confounders, such as comorbidities, oncological treatment status, or prior ED utilization. Third, a substantial proportion of presenting complaints were categorized as “other/unidentified,” reflecting limitations inherent to ED documentation and potentially reducing clinical granularity. Fourth, repeated visits by the same patient were treated as independent encounters, which may have introduced bias. Finally, differences observed between the two hospitals may reflect unmeasured structural factors, such as staffing levels, resource availability, or organizational practices.

Despite these limitations, this study provides clinically relevant insights into the patterns of ED utilization among oncology patients in a Central and Eastern European context, where data remain limited. By highlighting gaps in the recognition of palliative needs and patterns of care delivery, these findings may inform future research and support the development of more integrated, patient-centered models of emergency care.

Future studies should include longer observation periods, prospective designs, and broader clinical variables to better capture the complexity of oncology patients presenting to emergency departments. In particular, the development and validation of tools integrating acute triage with palliative assessment represent an important direction for further research.

## 5. Proposed System-Level Solutions

Based on our findings, several actionable strategies may improve the quality of care for oncology patients with palliative needs presenting to the ED:

1. **ED-Based Palliative Screening Tools**

The implementation of complementary screening instruments alongside standard triage scales may facilitate early identification of patients with advanced disease, frequent ED utilization, poor functional status, or refractory symptom burden. Such tools should be designed to identify palliative and end-of-life needs rather than replace acuity-based triage systems.

2. **Rapid Access to Palliative Care Consultation**

On-demand or trigger-based palliative care consultations within the ED may enable earlier goals-of-care discussions, optimized symptom management, and more appropriate disposition planning. Even brief palliative interventions in the emergency setting have been associated with improved patient-centered outcomes and reduced unnecessary hospital admissions.

3. **Structured ED-to-Hospice and ED-to-Community Pathways**

Establishing clear, standardized referral pathways from the ED to hospice or community-based palliative services may reduce barriers to timely end-of-life care. The ability to discharge patients directly from the ED to hospice or home-based palliative care, when clinically appropriate, is a key component of patient-centered emergency care.

4. **Education and Training Across Professional Career Stages**

Enhanced education in palliative medicine should be systematically integrated into emergency medicine training. This includes practical, decision-support algorithms for medical students and residents, as well as ongoing professional development for senior clinicians focused on prognostication, communication skills, and ethical decision-making at the end of life.

5. **Integration of Advance Care Planning Information**

Improved availability of advance directives, treatment limitation orders, and documented goals of care within ED information systems may facilitate timely, preference-concordant decision-making during acute clinical deterioration.

## 6. Implications for International Practice

The findings of this study underscore the role of the emergency department as a critical junction in the trajectory of serious illness. For many patients with advanced cancer, the ED encounter represents a pivotal moment at which the direction of care—life-prolonging versus comfort-focused—is implicitly determined.

Although this study was conducted within the Polish healthcare system, its implications extend well beyond national borders. Healthcare systems worldwide face increasing numbers of older patients with advanced malignancies, multimorbidity, and complex palliative needs. Integrating palliative principles into emergency care therefore represents a universal challenge, as well as an opportunity to improve the quality, equity, and appropriateness of end-of-life care.

Future research should prioritize prospective evaluation of ED-based palliative interventions, cross-national comparisons of care pathways, and the development of validated tools that bridge acute triage with palliative assessment. Reframing the ED not solely as a site of crisis management but also as a gateway to appropriate end-of-life care may help align treatment with patient values while reducing avoidable suffering.

## 7. Clinical Implications for Emergency and Palliative Care Practice

The results of this study have several practical implications for clinicians working at the interface of emergency and palliative care:

1. **Recognition of End-of-Life Trajectories**

Emergency clinicians should be supported in identifying indicators of advanced and terminal illness, including recurrent ED visits, declining functional status, refractory symptoms, and limited response to disease-directed therapies. Early recognition may prompt timely integration of palliative approaches alongside acute management.

2. **Symptom-Oriented and Goal-Concordant Care**

For patients with advanced cancer, ED care should prioritize rapid symptom relief and clarification of goals of care. Brief, structured discussions addressing patient preferences, treatment limitations, and acceptable outcomes may meaningfully influence clinical decision-making, even during time-limited encounters.

3. **Extending Triage Beyond Acuity Alone**

While standard triage systems such as the ESI remain essential for assessing immediate risk, they should be complemented by clinical judgment and palliative screening prompts to avoid under-recognition of significant suffering or end-of-life needs that may not meet classical emergency criteria.

4. **Facilitating Appropriate Disposition Decisions**

When clinically appropriate, direct referral from the ED to hospice or community-based palliative services should be considered a valid and patient-centered alternative to hospital admission. Clear institutional pathways are essential to enable such decisions safely and efficiently.

5. **Interdisciplinary Collaboration and Education**

Ongoing education in palliative care principles for ED staff—including physicians, nurses, and paramedics—can enhance confidence and competence in managing end-of-life situations. Close collaboration between emergency medicine, oncology, and palliative care teams is crucial to ensure continuity and quality of care.

## 8. Conclusions

Oncology patients presenting to EDs represent a vulnerable population with significant clinical needs. While triage systems identify acute risk, they may not fully capture palliative care needs. These findings suggest that improved integration of palliative care into emergency settings may enhance patient-centered outcomes.

## Figures and Tables

**Table 1:** Clinical outcomes among oncology patients presenting to two Emergency Departments (ED 1 and ED 2).

ED	Deaths (n)	Deaths (%)	Hospice Referrals (n)	Hospice Referrals (%)	Hospital Admissions (n)	Hospital Admissions (%)
ED 1	35	11	32	10	204	62
ED 2	4	2	2	1	114	52

**Table 2:** Distribution of cancer diagnoses among patients presenting to the Emergency Department. The table shows the four most common primary diagnoses (lung, breast, prostate, and colorectal cancers) and aggregated categories of other neoplastic diseases based on anatomical or pathological classification.

Cancer Group	Number of Patients	Percentage of Total (%)
**Other/Unidentified**	183	33.2
**Lung cancer**	55	10.0
**Breast cancer**	53	9.6
**Colorectal cancer**	44	8.0
**Gastrointestinal cancers**	42	7.6
**Prostate cancer**	40	7.2
**Unspecified neoplastic changes**	29	5.3
**Pancreatic cancer**	26	4.7
**Lymphomas and leukemias**	19	3.4
**Liver and biliary tract cancers**	18	3.3
**Genitourinary cancers**	15	2.7
**Central nervous system tumors**	12	2.2
**Gynecological cancers**	10	1.8
**Head and neck cancers**	2	0.4
**Skin cancers**	2	0.4
**Soft tissue sarcomas**	2	0.4

## Data Availability

The original contributions presented in this study are included in the article. Further inquiries can be directed to the corresponding author.

## References

[1] Majka E.S., Trueger N.S. (2023). Emergency Department Visits Among Patients with Cancer in the US. JAMA Netw. Open..

[2] Yilmaz S., Aryal K., King J., Bischof J.J., Hong A.S., Wood N., Rothberg B.E.G., Hudson M.F., Heinert S.W., Wattana M.K. (2025). Understanding oncologic emergencies and related emergency department visits and hospitalizations: A systematic review. BMC Emerg. Med..

[3] Lash R.S., Hong A.S., Bell J.F., Reed S.C., Pettit N. (2022). Recognizing the emergency department’s role in oncologic care: A review of the literature on unplanned acute care. Emerg. Cancer Care.

[4] Lee S.Y., Ro Y.S., Shin S.D., Moon S. (2021). Epidemiologic trends in cancer-related emergency department utilization in Korea from 2015 to 2019. Sci. Rep..

[5] Gould Rothberg B.E., Quest T.E., Yeung S.C.J., Pelosof L.C., Gerber D.E., Seltzer J.A., Bischof J.J., Thomas C.R., Akhter N., Mamtani M. (2022). Oncologic emergencies and urgencies: A comprehensive review. CA Cancer J. Clin..

[6] Mayer D.K., Travers D., Wyss A., Leak A., Waller A. (2011). Why do patients with cancer visit emergency departments? Results of a 2008 population study in North Carolina. J. Clin. Oncol..

[7] Amado-Tineo J.P., Oscanoa-Espinoza T., Vásquez-Alva R., Huari-Pastrana R., Delgado-Guay M.O. (2021). Emergency Department Use by Terminally Ill Patients: A Systematic Review. J. Pain Symptom Manag..

[8] Barbera L., Taylor C., Dudgeon D. (2010). Why do patients with cancer visit the emergency department near the end of life?. Can. Med. Assoc. J..

[9] Stone S.C., Mohanty S., Grudzen C.R., Shoenberger J., Asch S., Kubricek K., Lorenz K.A. (2011). Emergency medicine physicians’ perspectives of providing palliative care in an emergency department. J. Palliat. Med..

[10] Verhoef M.J., de Nijs E., Horeweg N., Fogteloo J., Heringhaus C., Jochems A., Fiocco M., van der Linden Y. (2020). Palliative care needs of advanced cancer patients in the emergency department at the end of life: An observational cohort study. Support. Care Cancer.

[11] Wallace E.M., Cooney M.C., Walsh J., Conroy M., Twomey F. (2013). Why do Palliative Care Patients Present to the Emergency Department? Avoidable or Unavoidable?. Am. J. Hosp. Palliat. Care.

[12] Dumnui N., Nagaviroj K., Anothaisintawee T. (2022). A study of the factors associated with emergency department visits in advanced cancer patients receiving palliative care. BMC Palliat. Care.

[13] Cairós-Ventura L.M., de Las Mercedes Novo-Muñoz M., Rodríguez-Gómez J.Á., Ortega-Benítez Á.M., Ortega-Barreda E.M., Aguirre-Jaime A. (2019). Validity and Reliability of the Emergency Severity Index in a Spanish Hospital. Int. J. Environ. Res. Public Health.

[14] Chi C.H., Huang C.M. (2006). Comparison of the Emergency Severity Index (ESI) and the Taiwan Triage System in Predicting Resource Utilization. J. Formos. Med. Assoc..

[15] Adler D., Abar B., Durham D.D., Bastani A., Bernstein S.L., Baugh C.W., Bischof J.J., Coyne C.J., Grudzen C.R., Henning D.J. (2019). Validation of the Emergency Severity Index (Version 4) for the Triage of Adult Emergency Department Patients with Active Cancer. J. Emerg. Med..

[16] Daemi A., Pourasghar F., Tabrizi J., Ala A., Jafarabadi M. (2016). Validity of the emergency severity index in predicting patient outcomes in a major emergency department. J. Nurs. Midwifery Sci..

[17] Bętkowska I. (2018). Emergencies in palliative care—clinical practice based on evidence. Med. Paliatywna W Prakt..

